# Dipththeria Antitoxin

**Published:** 1896-04-18

**Authors:** 


### Editor's Letter-Box.

### DIPHTHERIA ANTITOXIN.

Dr. R. Humphrey writes: I have read your article on "Diphtheria Antitoxin," and I think if medical men would disinfect the room in which the patient is in by simply burning a teaspoonful of sulphur every hour, directly the throat has an unhealthy ulceration or any membrane, they will find that the membrane rarely, if ever, attacks the larynx. I have now followed the treatment for sixteen years in a large country practice in Sussex, and have never had a bad case of laryngeal trouble since I began this treatment, but before I lost four or five cases from this cause. I may add I wrote an article in the *Midland Journal* about fifteen years ago on "The Treatment and Prevention of Diphtheria." If you wish I shall be pleased to answer any other questions on the subject.

